# Efficacy of a coordinated strategy for containment of multidrug-resistant Gram-negative bacteria carriage in a Neonatal Intensive Care Unit in the context of an active surveillance program

**DOI:** 10.1186/s13756-021-00902-1

**Published:** 2021-02-04

**Authors:** Laura Saporito, Giorgio Graziano, Federica Mescolo, Emanuele Amodio, Vincenzo Insinga, Grazia Rinaudo, Aurora Aleo, Celestino Bonura, Marcello Vitaliti, Giovanni Corsello, Francesco Vitale, Carmelo Massimo Maida, Mario Giuffrè

**Affiliations:** 1 Department of Health Promotion, Mother and Child Care, Internal Medicine and Medical Specialties, University of Palermo, Palermo, Italy; 2 Neonatology and Neonatal Intensive Care Unit, ARNAS Civico - Di Cristina - Benfratelli, Palermo, Italy

**Keywords:** Antimicrobial resistance, Active surveillance program, Multi-drug resistant gram-negative bacteria, Intervention strategy, Extended spectrum β lactamases producing klebsiella pneumoniae, Neonatal intensive care unit

## Abstract

**Background:**

Antimicrobial resistance in neonatal intensive care unit (NICU) patients is a threat, due to the frequent use of antimicrobial treatment and invasive devices in fragile babies. Since 2014 an active surveillance program of multidrug-resistant Gram-negative bacteria (MDR-GNB) carriage has been in place in the five NICUs of Palermo, Italy. In 2017 an increase in the prevalence of MDR-GNB, and in particular of extended-spectrum β-lactamases-producing *Klebsiella pneumoniae* (ESBL-KP), was observed in “Civico” hospital NICU.

**Aim:**

To assess the impact of a coordinated intervention strategy in achieving long-lasting reduction of MDR-GNB prevalence in the NICU.

**Methods:**

Rectal swabs were obtained monthly and processed to detect MDR-GNB using standard methods. MDR-GNB were characterized by pulsed-field gel electrophoresis (PFGE). Since November 2017 the following intervention measures were applied: (a) two-months intensification of sample collection; (b) stakeholders meetings; (c) improvement of prevention measures and antimicrobial policies.

**Findings:**

During the intensified microbiological surveillance MDR-GNB and ESBL-KP were detected in rectal swabs (34.8%; 23.2%), nasal swabs (24.6%; 14.5%), oral swabs (14.5%; 5.4%), milk samples (32.1%; 17.9%), pacifiers swabs (30.8%; 17.9%) and from sub-intensive room surfaces. Thirteen ESBL-KP strains isolated from clinical and environmental samples showed identical PFGE patterns. The prevalence of MDR-GNB and ESBL-KP carriage significantly decreased in the year after intervention compared to the previous year (20.6% vs 62.2%; *p* < 0.001 and 11.1% vs 57.8%; *p* < 0.001). MDR-GNB were not detected at all for three months and ESBL-KP for five months. Multivariate analysis of the principal exposure variables showed that admission in the post-intervention period significantly reduced the risk of MDR-GNB carriage (adj-OR = 0.21, 95% CI = 0.076–0.629; *p* < 0.001).

**Conclusions:**

MDR-GNB broadly circulate in NICU setting, they can colonize different body sites and spread through various vehicles. A coordinated strategy of multiple interventions with active cooperation between epidemiologists and clinicians in the NICU can effectively reduce their circulation and in particular the carriage of the most dangerous ESBL-KP strains.

## Introduction

The World Health Organization has declared antimicrobial resistance as one of the most dangerous public health threats of the last ten years with an increasing impact in the future [1].

Control and prevention of antimicrobial resistance and healthcare-related infections have been included in the Italian National Prevention Plan of Antimicrobial Resistance 2017–2020.

Neonatal Intensive Care Units (NICUs) are complex assistive settings, heavily burdened by antimicrobial resistance, due to the widespread use of antimicrobial treatments in critically ill patients exposed to invasive devices and procedures. In these settings, prevention and control of infections and multi-drug resistance can play a crucial role in the outcome of critically ill newborns with otherwise limited therapeutic options [2, 3]. Adherence by healthcare workers and parents to hand-washing protocols and other hygienic preventive measures in hospitals, particularly in intensive care units, is considered essential to managing the spread of healthcare-associated infections. [4].

Surveillance is commonly defined as the on-going and systematic collection, analysis and interpretation of health data essential to the planning, implementation and evaluation in public health practices [5]. Nowadays, active surveillance of infections or carriage by multidrug resistant organisms (MDROs) may be recognized as an effective tool for the early detection of unusual patterns of microbial pathogens in a specific health-care setting [6–8] and can be used to define the local epidemiology of MDROs and the relative antimicrobial resistance map in individual NICUs [8–11].

### Background

In accordance to internationally recognized prevention objectives [12], the University Hospital in Palermo, Italy, started an active surveillance program of MDRO carriage in the NICU in June 2009 [13–15]. In 2014 this program was extended to the other four NICUs in the metropolitan area in order to define antimicrobial resistance patterns in different settings, and to monitor eventual microbial circulation related to patient movements inside the network of the local health system [16, 17].

The main focus of the program, which is still ongoing, is the epidemiological analysis of MDROs circulation, seasonal variability, associated risk factors, molecular typing of isolated bacteria and evaluation of antimicrobial resistance related to bacterial carriage. This active surveillance program involves an epidemiological team with its laboratory, a neonatal team, and NICU healthcare providers. Each stakeholder participates in regular meetings, in order to identify shared goals and methodology and to provide periodic feedback of their activities. Collection of nasal and rectal swabs from each hospitalized newborn is performed every 4 weeks (for the purposes of this study, each 4-week interval was defined as a month) in every NICU. Specimens are processed in order to identify carriage by MDROs. Complete figures and trends of microbial isolations are registered and periodically reported to healthcare providers, together with the results of the in vitro antimicrobial sensitivity testing. The ultimate purpose is prevention and control of infectious diseases through the early identification of new carriage clusters or changes in the time-line and pattern of colonization in a specific setting.

During the first four years of the program, we observed a higher prevalence of multi-drug resistant Gram-negative bacteria (MDR-GNB) carriage in the "Civico" hospital NICU compared to the other NICUs in Palermo. A thorough examination of the annual prevalence of MDR-GNB carriage in rectal swabs in this health-care setting pinpointed a high percentage of MDR-GNB colonized newborns in 2014 and an increasing trend in 2015 (Table 1).

**Table 1:** Number and percentage of rectal samples positive for MDR-GNB, ESBL-producing GNB and ESBL-KP during four years surveillance in “Civico” Hospital NICU

period of surveillance	2014 (Feb—Dec)	2015 (Jan–Dec)	2016 (Jan–Dec)	2017 (Jan–Oct)
tested samples, n	180	184	164	160
MDR-GNB, n (%)	97 (53.9)	126 (68.5)^a^	71 (43.3)^a,b^	112 (70.0)^a,c^
ESBL, n (%)	92 (51.1)	102 (55.4)	61 (37.2)^a,b^	104 (65.0)^a,c^
ESBL-KP, n (%)	91(50.6)	43 (23.4)^a^	40 (24.4)^a^	104 (65.0)^a,b,c^

^a^*p* < 0.05 with respect to 2014

^b^*p* < 0.05 with respect to 2015

^c^*p* < 0.05 with respect to 2016

Based on this evidence, few additional episodic control measures were performed: an extraordinary collection of rectal swabs in November 2015 and a meeting with all health-care workers in January 2016 to inform them about the worrying colonization data and trends and to reinforce good clinical practices for infection control and prevention.

Despite an initial significant reduction of MDR-GNB carriage in 2016 (*p* < 0.001), the annual prevalence again showed an increase in 2017 (*p* < 0.001, Table 1), mainly involving Extended-spectrum β-lactamases-producing *Klebsiella pneumoniae* (ESBL-KP). This evidence led to the introduction in November 2017 of a more effective and long-lasting intervention to reinforce the implementation of the measures already adopted.

### Aim of the study

The aim of this study was to quantify the impact of a coordinated intervention strategy in achieving a more effective and long-lasting reduction of MDR-GNB colonization prevalence in “Civico” hospital NICU. This was done by comparing data from the “pre-intervention” period (from November 2016 to October 2017) with the “post-intervention” period (from November 2017 to October 2019) and evaluating the role of multiple clinical risk factors.

## Patients and methods

### Setting and population

The “Civico” hospital NICU is set in the adult hospital campus and includes two adjacent open spaces of 60 and 40 square meters respectively. The NICU has 16 beds, of which 8 intensive and 8 sub-intensive. A hand-washing sink is located at each entrance. Around 370 term and preterm newborns are admitted annually.

Each area, intensive and sub-intensive, has two dedicated neonatologists and two dedicated nurses, while another nurse is responsible for feeding-preparation.

All newborns included in our monthly surveillance program between November 2016 and October 2019 were enrolled in this study. Rectal swabs for detection of MDR-GNB carriage were collected every 4 weeks from all newborns in the NICU, regardless of any clinical or laboratory signs of infection. Carriage was defined as a positive culture of MDR-GNB from at least one rectal swab collected during the NICU stay.

Since November 2017, a strategy of multiple coordinated intervention measures was introduced to reduce the prevalence of MDR-GNB carriage.

### Description of intervention measures

The intervention strategy included:

#### Intensification of sample collection

Microbiological surveillance was reinforced for 2 months (11/27/2017—01/23/2018) as follows:

- additional weekly samplings of rectal, nasal and oral mucosa swabs, swabs of devices and material in direct contact with each newborn (feeding bottles and pacifiers, and remnant milk after newborn feeding) for the first month and every four weeks afterwards;
- environmental samplings (room surfaces including milk preparation room, healthcare workers hands and stethoscopes) at the beginning of the intervention and after two months. In February 2018 monthly sample collection reverted to the previous surveillance with only rectal swabs.

#### Stakeholders weekly meetings

During the first two months of intervention, weekly meetings of NICU healthcare workers were held with experts from the surveillance team, sharing surveillance program results, pinpointing adherence of healthcare workers to standard precautions and discussing possible critical issues and preventive strategies. Subsequently, these meetings were scheduled monthly and involved only the NICU staff.

#### Improvement of prevention measures

The following changes in NICU organization and patient management were introduced:

- a new standard protocol for antimicrobial therapy was approved by the Hospital Health Management. The new guidelines standardized the timing for starting and stopping therapy in suspected sepsis, the duration of therapy in confirmed infections and sepsis, and the stopping of therapy after the first negative culture [18];
- hand-washing sensitization posters for caregivers and parents were placed in all rooms;
- contaminated devices were replaced with clean ones after feeding;
- introduction of bundles for common procedures, such as blood-sample collection, diaper change, milk preparation or fridge sanitation.

### Evaluation of the impact of intervention measures

The prevalence of MDR-GNB, ESBL-producing GNB, ESBL-KP carriage was assessed and compared in two groups: newborns admitted in the pre-intervention period and newborns admitted in the post-intervention period.

In addition, in order to better evaluate the impact of the intervention measures and identify confounding factors that could impact MDR-GNB carriage, we conducted a quasi-experimental study comparing the clinical features in two subgroups of patients: intervention population and control population.

Inclusion criteria were admission in the NICU and enrollment in the surveillance program for MDR-GNB from November 2017 to March 2018 for the intervention population, and from November 2016 to March 2017 for the control population.

Exclusion criteria were:

- clinical records not available;
- outborn patients with MDR-GNB colonization in the first rectal swab (we do not perform rectal swab on NICU admission so we could not discriminate if MDR-GNB carriage was already present before the admission in the ward);
- patients with a positive rectal swab before the implementation of preventive measures (November 28th 2017).

The outcome variable was detection of MDR-GNB in at least one rectal swab obtained for the surveillance program during the observation period of risk factors exposure.

The observational period was defined in colonized patients as the span of time between NICU admission and the date of the first positive rectal swab, and in non-colonized patients as the span of time between NICU admission and the last rectal swab obtained.

We analysed the following clinical characteristics: type of delivery, sex, gestational age, birth weight, APGAR score at 5′, presence of malformations, feeding (breast milk and/or formulas), use of nasogastric tube, parenteral nutrition, use of invasive devices (central and peripheral venous access), invasive or non-invasive ventilation, surgical treatment, use of antimicrobials and hospital discharge (alive, dead or moved to another hospital).

### Collection of samples and microbiological analysis

Collected samples were enriched in liquid cultures (Brain Heart Infusion, Oxoid) for 24 h at 37 °C, then plated in McConkey Agar with three antimicrobial discs (amoxicillin-clavulanate 30 μg, meropenem 10 μg, ceftazidime 30 μg) to detect multi-drug resistant bacteria [13]. After overnight incubation, suspected MDR colonies were isolated for identification (standard biochemical methods), susceptibility testing and ESBL detection according with the European Committee on Antimicrobial Susceptibility Testing (EUCAST) guidelines [19–21].

MDR-GNB were defined as Gram-negative bacteria non-responders in vitro to at least three different classes of antimicrobials under testing (aminopenicillins, third-generation cephalosporins, monobactams, aminoglycosides and carbapenems).

Molecular characterization of MDR-GNB was performed using pulsed-field gel electrophoresis (PFGE) after DNA-cutting with restriction enzymes and electrophoretic profiles were interpreted according to standard procedures [18, 22, 23].

### Statistical analysis

Prevalence of MDR-GNB carriage, in relation to patient characteristics, procedures and clinical outcomes were compared using the chi-square test or Fisher test for categorical variables and Student’s t-test or the Mann–Whitney U test for continuous variables.

A multivariate analysis by backward stepwise logistic regression was carried out to determine variables significantly associated with MDR-GNB carriage. All variables that did differ between subjects with MDR-GNB carriage (*p* < 0.10) were initially entered in the model, and the least significant variables were removed one at a time. Goodness of fit of the logistic models was assessed using the Hosmer and Lemeshow test. Several multiple logistic regression models were tested in order to determine the most significant and simplest model with the best available fit for the data.

All significance tests were two-tailed, and *p* < 0.05 was considered significant.

Statistical analysis was carried out by using the R statistical software package (version 3.6.1) and Microsoft Excel 2010.

## Results

### Intensified microbiological surveillance

**Fig. 1:**
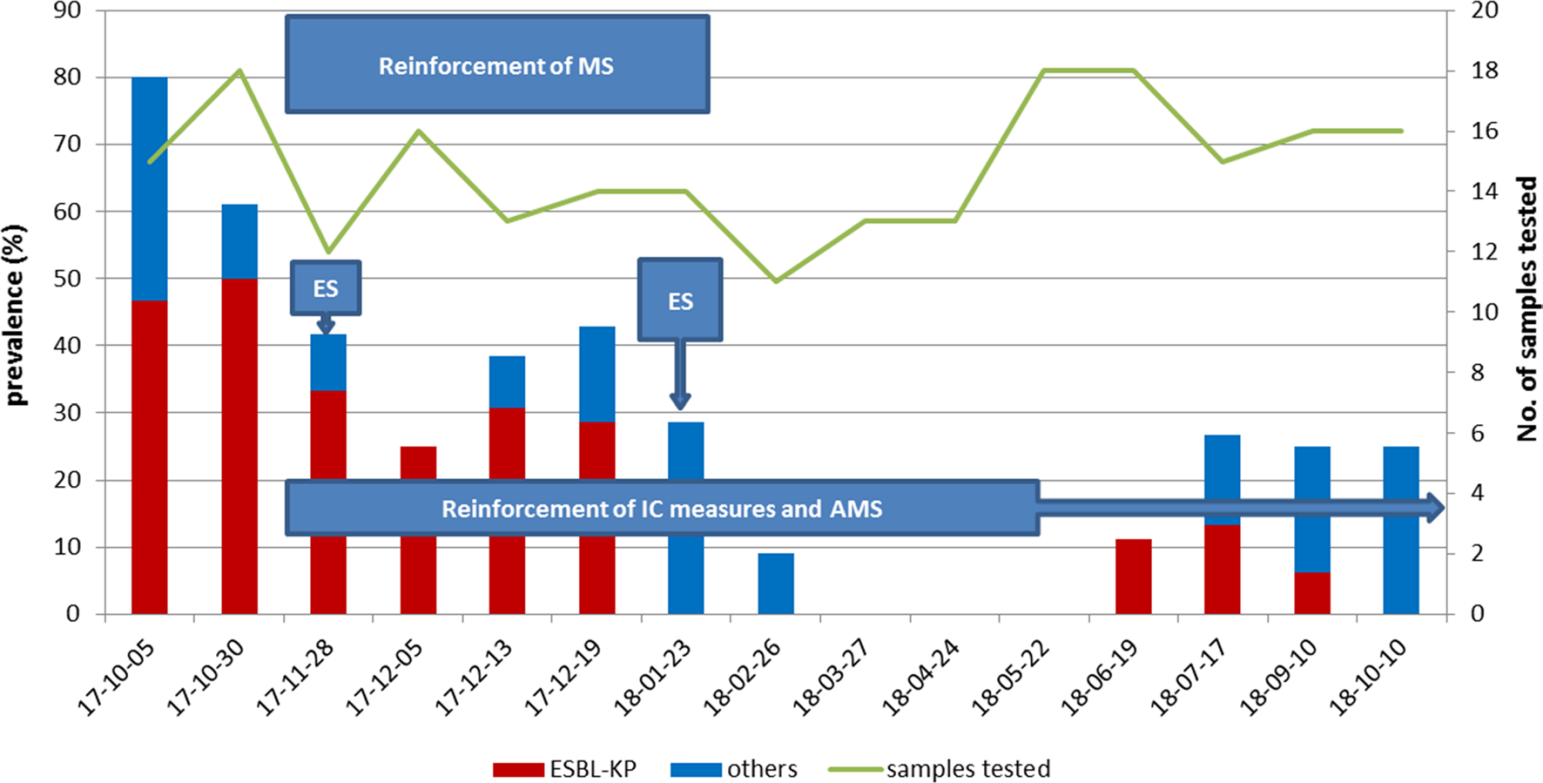
Temporal trend of the prevalence of MDR-GNB rectal carriage and combined measures of infection control adopted. Column color shows the proportion of ESBL-KP positive samples (red) vs other MDR-GNB (blue). Green line represents the number of samples tested. AMS = antimicrobial stewardship; ES = environmental sampling; IC = infection control; MS = microbiological surveillance

During the intensified microbiological surveillance (performed as described above from November 28 2017 to January 23 2018) 69 rectal swabs, 69 nasal swabs, 55 oral swabs, 39 samples from feeding bottles and pacifiers and 28 milk samples were collected. MDR-GNB were detected from 24 rectal swabs (34.8%), 17 nasal swabs (24.6%), 8 swabs from oral mucosa (14.5%); moreover, 9 milk samples (32.1%) and 12 pacifiers swabs (30.8%) were positive for MDR-GNB. ESBL-KP was detected from 16 rectal swabs (23.2%), 10 nasal swabs (14.5%), 3 swabs from oral mucosa (5.4%); 5 milk samples (17.9%) and 7 pacifiers swabs (17.9%) were positive for ESBL-KP. The results indicate that MDR-GNB were significantly reduced during the post- intervention months and ESBL-KP was not detected in the ward from January 23 to June 2018. Temporal trends of MDR-GNB and ESBL-KP rectal carriage in relation to the intervention measures adopted are shown in Fig. 1.

Environmental testing yielded 104 samples: 45 NICU surface samples, 23 sub-intensive room surface samples, 11 specimens from feeding-preparing surfaces, 15 from caregivers’ hands, 5 from stethoscopes, 5 from baby cots, 2 from laminar-flow hoods. One powder and one liquid feeding formula sample were also analysed.

MDR-GNB isolates were detected in one sample from the sub-intensive room (ESBL-KP) on the first environmental sampling day and in one sample from the feeding-preparing room (*Stenotrophomonas maltophilia*) on the second environmental sampling day. In the same data set, non-MDR *Pseudomonas aeruginosa* was isolated in two samples from the sub-intensive and feeding-preparing room. No MDR-GNB were isolated from healthcare workers hands.

**Fig. 2:**
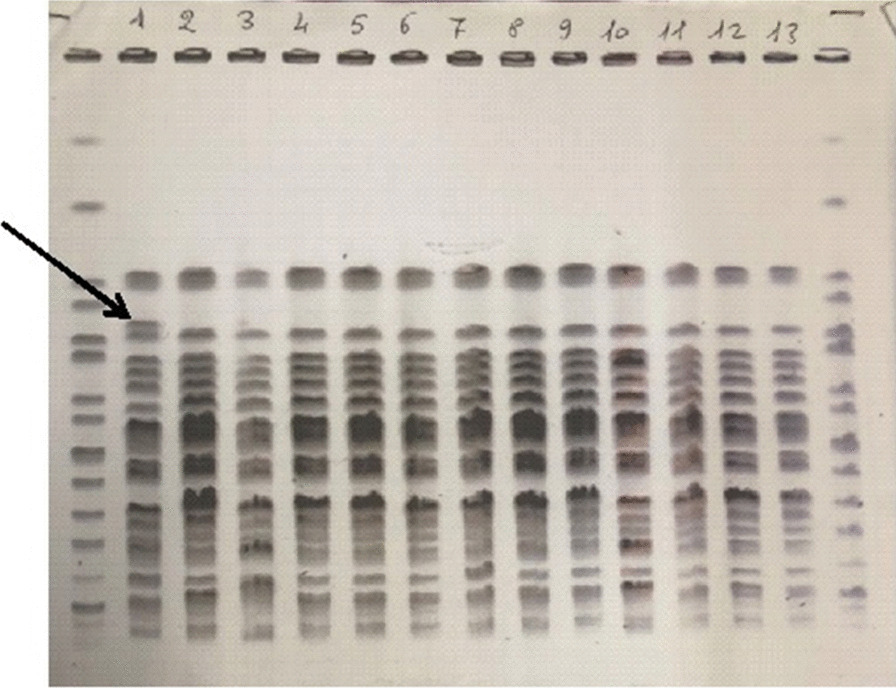
PFGE profile of 13 ESBL-KP strains isolated from rectal swab [1–4, 6–10], oral swab [3–7], nasal swab [13], soothers [2–5, 8, 9], milk [11], belonging to 5 patients and one environmental sample obtained from the changing table of the intermediate care room [12]. Samples 2 to 13 show identical pulsotype. Sample 1 differs by the presence of one band of about 350 Kb (arrow)

Thirteen ESBL-KP strains isolated from rectal, nasal, oral swabs, milk, soothers and environmental surfaces were submitted to molecular characterization by PFGE. Analysis of electrophoretic profiles showed identical or closely related patterns suggesting a common origin for all tested strains (Fig. 2).

### Comparison between pre- and post-intervention periods

In the total study period (November 2016—October 2019) 419 patients were enrolled in the surveillance, 142 in the pre-intervention period (November 2016—October 2017), 143 and 134 respectively in the first and in the second year after the intervention (November 2017—October 2019). A mean of 15 (SD = 2.76) patients were screened each month during the pre-intervention period. There was no significant difference in the post-intervention period when 14.9 patients (SD = 2.61) were tested each month (*p* = 0.96).

**Fig. 3:**
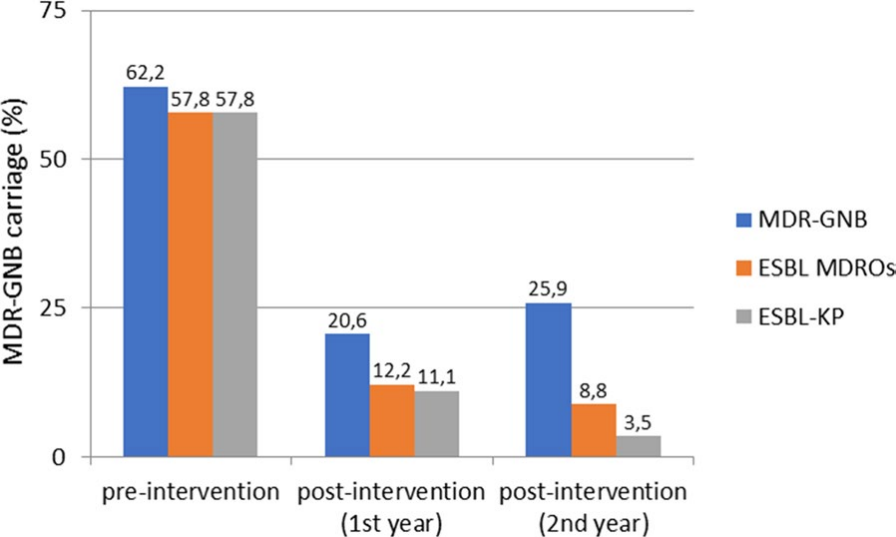
MDR-GNB carriages in 539 rectal swabs collected in the pre- and post-intervention periods showing a significative and persistent decrease in prevalence of all MDR-GNB positives, and in particular of ESBL-KP positive (*p* < 0.001 between pre-intervention and 1^st^ year post-intervention; *p* = 0.006 between 1st and 2nd year post-intervention)

A total of 539 rectal swabs were collected and analysed. In the pre-intervention period 180 rectal swabs were analysed: MDR-GNB were detected from 112/180 (62.2%) rectal swabs and, in particular, 104/180 (57.8%) were ESBL-KP. In the first year of the post-intervention period 189 rectal swabs were analysed: MDR-GNB were detected from 39/189 (20.6%) rectal swabs and, in particular, 21/189 (11.1%) were ESBL-KP, while 2/189 (1.1%) were other ESBL-producing GNB. Carriage of ESBL-KP accounted for 92.8% (104/112) of all MDR-GNB isolated in the pre-intervention period and for 53.8% (21/39) of those isolated in the first year of the post-intervention period. Prevalence of MDR-GNB, ESBL-producing GNB and ESBL-KP carriage between the two periods was significantly different (*p* < 0.001). Chi square for trend was also significant (*p* < 0.001). In the second year of the post-intervention period (November 2018—October 2019) 170 samples were analysed. Prevalence of MDR-GNB carriage remained low (MDR-GNB 44/170, 25.9%; ESBL-producing GNB 15/170, 8.8%; ESBL-KP 6/170, 3.5%). No significant differences were observed in the prevalence of MDR-GNB and ESBL-GNB with the previous year (*p* = 0.24 and *p* = 0.30, respectively). Prevalence of ESBL-KP was significantly lower in the second post-intervention year (*p* = 0.006). Trends of MDR-GNB carriage in the pre- and post-intervention periods are shown in Fig. 3.

### Quasi-experimental study: comparison between intervention population and control population

**Fig. 4:**
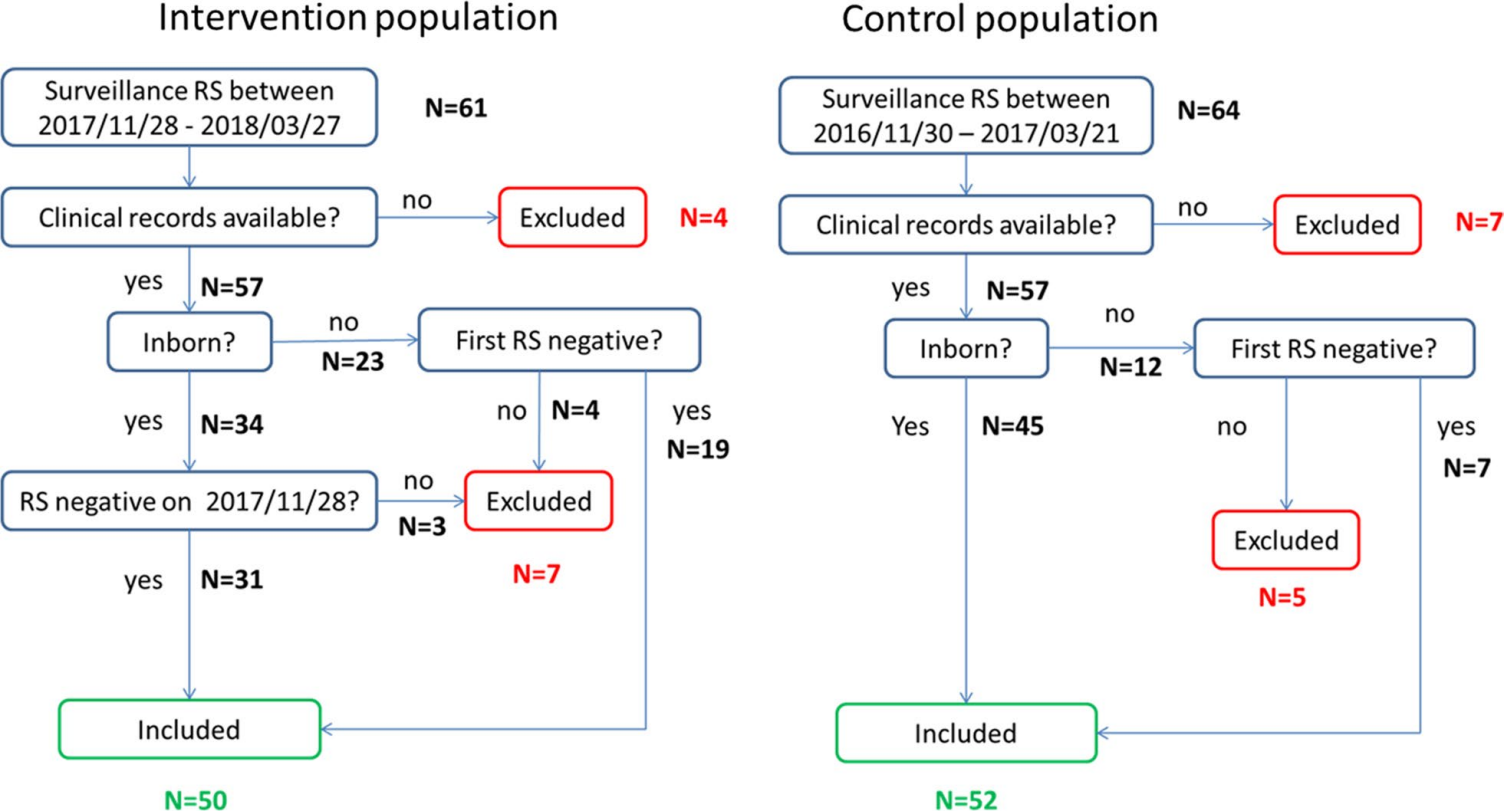
Algorithm for selection of intervention population and control population. RS = rectal swab

One-hundred and two patients fulfilled the criteria to be included in the quasi-experimental study: 50 patients admitted from November 2017 to March 2018 (intervention population) and 52 patients admitted from November 2016 to March 2017 (control population), according to the inclusion/exclusion algorithm (Fig. 4).

Characteristics of patients, medical devices and antimicrobial treatment are summarized in Table 2.

**Table 2:** Comparison between different variables in intervention population and control population in quasi-experimental study population

Variable	All patients (n = 102)	Intervention population (n = 50)	Control population (n = 52)	*p*
*Characteristics of patients*				
Male gender, n (%)	54 (52.9%)	29 (58%)	25 (48.1%)	0.31
Twin birth, n (%)	15 (15.5%)	6 (12.5%)	9 (18.4%)	0.42
Inborn, n (%)	76 (74.5%)	31 (62%)	45 (86.5%)	**0.004**
Admission to NICU > 24 h after birth, n (%)	9 (8.9%)	7 (14%)	2 (3.9%)	0.09
Birth through caesarean section, n (%)	65 (65%)	30 (60%)	35 (70%)	0.29
Gestational age, median (IQR), wks	34.5 (32-38)	36.5 (33-39)	33 (32-37)	**0.014**
Preterm birth (< 37wk), n (%)	63 (61.8%)	25 (50%)	38 (73.1%)	**0.02**
Birth weight, mean (SD), g	2234 (865)	2509 (844)	1980 (811)	**0.002**
Apgar score at 5 min, median (IQR)	9 (8-10)	9 (8-10)	9 (8-10)	0.71
Malformation, n (%)	11 (10.8%)	6 (12%)	5 (9.6%)	0.70
*Nutrition and devices*				
Breast milk feeding, n (%)	75 (73.5%)	32 (64%)	43 (82.7%)	**0.03**
Formula feeding, n (%)	101 (99%)	49 (98%)	52 (100%)	0.49
Nasogastric tube, n (%)	43 (42.2%)	19 (38%)	24 (46.1%)	0.40
Parenteral nutrition, n (%)	60 (58.8%)	29 (58%)	31 (59.6%)	0.87
Central venous access device, n (%)	44 (43.1%)	20 (40%)	24 (46.1%)	0.53
Peripheral venous access device, n (%)	81 (79.4%)	37 (74%)	44 (84.6%)	0.18
Endotracheal tube, n (%)	19 (18.6%)	6 (12%)	13 (25%)	0.09
Noninvasive ventilation, n (%)	41 (40.2%)	22 (44%)	19 (36.5%)	0.44
Surgical procedure, n (%)	3 (2.9%)	2 (4%)	1 (1.9%)	0.61
*Antimicrobial therapy*				
Antibiotics, any, n (%)	49 (48%)	21 (42%)	28 (53.8%)	0.23
Ampicillin, n (%)	24 (23.5%)	16 (32%)	8 (15.4%)	0.048
Ampicillin—Sulbactam, n (%)	11 (10.8%)	0	11 (21.1%)	** < 0.001**
Cephalosporines, n (%)	21 (20.6%)	7 (14%)	14 (26.9%)	0.11
Carbapenems, n (%)	18 (17.6%)	9 (18%)	9 (17.3%)	0.93
Amikacin, n (%)	32 (31.4%)	16 (32%)	16 (30.8%)	0.89
Glycopeptides, n (%)	17 (16.7%)	8 (16%)	9 (17.3%)	0.86
Metronidazole, n (%)	8 (7.8%)	5 (10%)	3 (5.8%)	0.48
Fluconazole, n (%)	20 (19.6%)	6 (12%)	14 (26.9%)	0.06
*Rectal swab colonization*				
MDR-GNB, n (%)	28 (27.4%)	6 (12%)	22 (42.3%)	** < 0,001**
ESBL, n (%), all ESBL-KP	23 (22.5%)	1 (2%)	22 (42.3%)	** < 0,001**

Statistically significant values (*p* < 0.05) are reported in bold characters

Among the 102 patients, 54 (52.9%) were male, 15 (15.5%) were twins, 76 (74.5%) were inborn. Caesarean section was the most frequent type of delivery (65%), median gestational age was 34 weeks and 61.8% of babies were born preterm. Mean birth weight was 2234 g and median APGAR score was 9. Eleven (10.8%) patients had some kind of malformations. Nine (8.9%) patients were admitted to the NICU more than 24 h after birth. Almost all newborns (99%) were given formula complemented by breast milk in 73.5% of cases. Use of medical devices ranged between 18.6% for endotracheal tube and 79.4% for peripheral venous catheter. Three (2.9%) patients had surgery during their hospitalization. Antimicrobial therapy was administered in 48% of patients. Amikacin (31.4%) and ampicillin (23.5%) were the most frequently used drugs. MDR-GNB carriage affected 28 patients (27.4%). In particular, all ESBL-producing GNB were ESBL-KP (23 patients, 22.5%). *Enterobacter* spp. and *Escherichia coli* carriage accounted for 3.9% and 1% of patients respectively. Infection was diagnosed in 28 patients (27.7%) and 1 (1%) patient died. Mean duration of stay was 24 days.

Distribution of some characteristics was significantly different between control and intervention population (Table 2). In particular, the proportion of inborn, preterm infants and breast milk feeding was significantly higher in the control population (*p* = 0.004, *p* = 0.02 and *p* = 0.03 respectively), while birth weight was significantly lower (*p* = 0.002). Mean duration of hospital stay markedly decreased from 28.4 days (SD 17.3) in the control population to 20 days (SD 19.4) in the intervention population (*p* = 0.02). In the intervention population ampicillin-sulbactam was replaced by ampicillin.

Prevalence of MDR-GNB carriage was significantly lower in the intervention population compared with controls (12% vs. 42.3%, *p* < 0.001), namely ESBL-KP positivity on rectal swabs showed a significant reduction (2% vs. 42.3%, *p* < 0.001).

Multivariate analysis showed that the intervention population had a significantly reduced risk of MDR-GNB carriage compared to the control population (adj-OR = 0.21, 95% CI 0.076–0.629; *p* = 0.0047), whereas breast milk feeding was associated with an increased risk of MDR-GNB carriage (adj-OR = 11.9, 95% CI = 1.49–95.9; *p *value = 0.019) (Table 3).

**Table 3:** Association between MDR-GNB carriage and multiple exposure variables in quasi-experimental study population

Exposure variable	OR (95% CI)	*p *Value	Risk of MDR-GNB carriage (adj-OR – 95% CI)	Adj-p*
*Sex, ref. F*	1.26 (0.52–3.10)	0.60		
Admission in post-intervention period, ref. No	0.19 (0.06–0.51)	0.001	0.21 (0.076–0.629)	0.0047
Inborn, ref. No	6.1 (1.5–41.3)	0.02		
Caesarean delivery, ref. No	1.9 (0.72–5.4)	0.19		
Surgical intervention, ref. No	5.6 (0.49–64.5)	0.18		
Gestational age, per week increase	0.87 (0.77–0.98)	0.023		
Birth weight, per 100 gr increase	0.98 (0.97–0.99)	0.027		
Breast milk feeding, ref. No	14.4 (2.5–313.5)	0.001	11.9 (1.49–95.9)	0.019
Nasogastric tube, ref. No	2.82 (1.15–7.1)	0.02		
Endotracheal tube, ref. No	1.7 (0.56–4.9)	0.31		
Ampicillin, ref. No	0.45 (0.12–1.4)	0.18		
Ampicillin-sulbactam, ref. No	3.7 (0.98–14.4)	0.07		
Cephalosporins, ref. No	3.1 (1.1–8.8)	0.02		
Carbapenems, ref. No	1.02 (0.3–3.13)	1		
Glycopeptides, ref. No	1.12 (0.35–3.5)	1		
Fluconazole, ref. No	2.7 (0.94–7.6)	0.051		
Duration of hospital stay before colonization, per day increase	0.96 (0.92–0.99)	0.04		

^*^Hosmer–Lemeshow Goodness-of-Fit Test *p *value = 0.94

## Discussion

After the evidence of a high MDR-GNB carriage in “Civico” NICU during the first two years of surveillance program, an episodic control strategy was implemented through an extraordinary sampling of patients and an informational meeting involving all healthcare workers of the ward in order to reinforce adherence to hand hygiene and good clinical practices. These measures resulted in a short-term decrease of MDR-GNB carriage prevalence from 68.5% in 2015 to 43.3% in 2016. However, after few months, the prevalence of MDR-GNB carriage increased again to reach 70% in 2017 (Table 1). A high prevalence of MDR-GNB carriage has also been reported from Ecuador (56%), Philippines (61%) and Hungary (> 50%) [24, 25]. Nevertheless, differences in local epidemiology, logistics and hospital organization must be considered. In our setting, the contextual rapid increase in ESBL-KP carriage, that accounted for most MDR-GNB identified, suggested the need for a more structured and permanent intervention to achieve long-lasting containment of carriage prevalence. This intervention strategy included the intensification of sample collection with extraordinary clinical and environmental samples, frequent stakeholders meetings and an improvement in prevention measures such as protocols for the correct use of antibiotic therapy, sensitization to hand-washing, implementation of checklists for common and invasive procedure management.

The impact of this multiple and coordinated intervention strategy for the reduction of MDR-GNB carriage was statistically significant and sustained (especially in regard to ESBL-KP) even in the second year after intervention, with a further reduction of ESBL-KP prevalence despite a modest increase of MDR-GNB carriage (Fig. 3). It should be noted that, since the beginning of the surveillance program, rectal samples were collected every 4 weeks from all newborns in the NICU, regardless of any clinical or laboratory signs of infection; therefore, previously colonized patients were sampled again if still present in the NICU 4 weeks later. The number of patients and samples tested was similar in the pre-intervention period and in the first and second year after the intervention and the mean number of samples collected from each patient was 1.3, both in the pre-intervention and in the post-intervention period. Thus, we are confident that the higher rate of MDR-GNB carriage in the pre-intervention period is not the result of repeated sampling of a cohort of colonized babies.

The two months intensified surveillance of patients together with microbiological analysis of surfaces and healthcare workers’ hands was useful for strictly monitoring carriage trends, tracing transmission roots and enhancing the adherence to preventive measures, most importantly hand hygiene in the “five key moments” as suggested by WHO [26–28]. During this period, MDR-GNB were detected not only from rectal swabs, but also from nasal and oral mucosa swabs. In addition, feeding bottles, pacifiers and milk samples were analysed because, when contaminated during feeding, they can represent an additional source of MDR-GNB environmental spreading. The role of cross-transmission of MDR-GNB was confirmed by the presence of identical or closely-related ESBL-KP strains in rectal, oral, nasal swabs, milk and environmental samples (Fig. 2). The evidence of MDR-GNB spread by saliva and milk increased the awareness of physicians and nurses and the adherence to hand hygiene before and after milk administration and led to the immediate substitution of contaminated devices after feeding [29]. In our experience, the finding of environmental contamination by intestinal bacteria highlighted the vital role of healthcare workers in the prevention of such spreading and prompted the implementation of a detailed procedure for diaper change and a better compliance to correct actions. Environmental contamination by ESBL-KP in the NICU has been reported by Szél and colleagues and was associated with high prevalence of MDR-GNB carriage and infection [24]. Successful elimination of ESBL-producing nosocomial bacteria was accomplished with the implementation of a multidisciplinary intervention based on reduction of invasive procedures, changes of the antibiotic policies, microbiological screening at short intervals, progressive feeding, safer bathing protocol, staff hand hygiene training and continuous monitoring of the number of newly infected and newly colonized patients. This interdisciplinary approach aligns with ours for most of the measures taken and our results are comparable. In 2018 in Montpellier, a hospital surveillance program revealed an outbreak of ESBL-KP infection/colonization with incubators as the probable pathogen reservoir. ESBL-producing strains from 19 patients shared the same molecular profile among themselves and with a strain isolated from an incubator after cleaning. In accordance with our findings, the introduction of new preventive hygiene measures stopped the outbreak, thus highlighting the fundamental role of environmental colonization management [30].

The role of MDR-GNB intestinal carriage as a risk factor for infection has been reported in several studies [29, 31, 32]. For example, colonization by ESBL-producing GNB was a risk factor for developing ESBL infections in paediatric cardiac surgery patients [33]. Above all, the cross-transmission of colonization, is a sign of poor adherence to infection prevention measures, therefore the prevalence of colonization could be used as an indicator of health workers' compliance with standard and contact precautions in patient care. Furthermore, carriage certainly represents a potential source of dissemination of microorganisms from colonized patients to other NICUs or other paediatric and community health facilities [34]. Recent studies have shown the persistence of MDR-GNB colonization even up to 2–5 years after NICU discharge, thus emphasizing the impact of the problem [35].

Our analysis considers the role of a whole set of control measures performed at the same time so that we are not able to identify the single contribution of each group of actions (intensification of sample collection, improvement of prevention measures or stakeholders weekly meeting) on the reduction of MDR-GNB and ESBL-KP carriage. Different studies have analysed the impact of single measures on microbial colonization or infection. The correct management of central venous access proved to be effective against related infections [36]. Antimicrobial stewardship for the correct use of antibiotics in term of doses, duration of therapy and administration route is a key point for prevention and control of drug-resistance [37–39]. In other studies appropriate audits of outcomes and feedback to stakeholders have been included among essential infection prevention strategies in the paediatric population [40]. Intensification of microbiological surveillance has been used as a strategy to contain ESBL-KP outbreaks [30].

The quasi-experimental analysis was carried out in order to determine if differences in the clinical characteristics of patients could have had a confounding effect in the reduction of MDR-GNB carriage after introduction of coordinated intervention measures. Clearly defined selection criteria were used for both intervention and control populations in order to minimize selection bias. Possible confounders related to different structural, organizational and seasonal settings were ruled out by choosing as controls a group of patients admitted in the same hospital ward in the same season of the preceding year and cared for by the same healthcare personnel. Prevalence of MDR-GNB and ESBL-KP carriage was significantly reduced in the intervention population compared to controls (12% vs 42.3% and 2% vs 42.3%, respectively). The two populations were compared in respect to clinical features, use of medical devices (invasive and non-invasive) and type of treatment; differences for specific variables were observed and statistically investigated (Table 2). Gestational age, birth weight, being inborn, type of feeding, antibiotics and length of stay in NICU were variables associated to MDR-GNB carriage at univariate analysis and were included in the multivariate model in order to analyse their possible contribution to the global risk of colonization. The risk of colonization by MDR-GNB in patients admitted during the intervention period was significantly decreased nearly fifth fold compared to that observed in the patients admitted during the control period. In addition, multivariate analysis confirmed the significant role of the introduction of coordinated intervention measures in reducing bacterial circulation, regardless of patient characteristics and procedures (Table 3).

Being inborn seemed to increase the risk of colonization (OR = 1.90) even if this association was not significant (0.55).

Cassettari et al*.* observed a possible protective role of breastfeeding against ESBL-KP colonization in newborns [41], but our data did not support this evidence (adj-OR = 11.9, *p* = 0.019), so the role of this factor needs to be further elucidated. The intrinsic protective role of human milk against infection is reported in several studies [42–44] but some authors have reported an increased risk of colonization in breast milk fed newborns in intensive care settings [45, 46], which is in accord with our data. Possible biases in our population were related to the large amount of newborns fed with both maternal milk and formula. In addition, the handling and storage of expressed breast milk before administration should be further evaluated.

Previous studies reported the role of low gestational age and birth weight, mechanical ventilation, parenteral nutrition, invasive devices and antibiotics use as risk factors associated to MDR-GNB carriage [25, 34, 44, 46]. In our experience these associations were confirmed but were not significant, probably due to the small number of patients examined. The risk of colonization increased with lower gestational age and with the use of nasogastric tube (OR = 0.73, *p* = 0.09 and OR = 2.68, *p* = 0.22 respectively). Moreover, lower birth weight was not significant probably because the mean weight in our population was high. First-line empiric antibiotic treatment was significantly different between the two groups. In particular, ampicillin-sulbactam was replaced by ampicillin in the intervention period, as suggested by international guidelines [47]. Univariate analysis showed a significant association between the use of cephalosporins and the risk of MDR-GNB carriage (OR = 1.54, *p* = 0.02), however, this association was not confirmed by multivariate analysis (*p* = 0.68).

Our study did have some limitations. The complexity of the preventive intervention did not allow for evaluating the specific contribution of each measure implemented towards the reduction of MDR-GNB carriage. Some patients were excluded from the analysis because of the lack of clinical records; therefore we cannot know if their inclusion would have resulted in the identification of some other risk factors associated with MDR-GNB carriage. Moreover, we did not perform any screening on the mothers, so we could not rule out this likely source of colonization, as previously demonstrated by Danino et al. [48]. Finally, in our five-year active surveillance program (2014–2019) the collection of rectal swabs was scheduled only every four weeks due to organizational needs, and included all the newborns in the NICU at the time of sample collection. As a consequence of this schedule, newborns hospitalized in the period between monthly samplings were not tested.

However, we believe that a network-based approach is essential for managing the circulation of multi-resistant pathogens, because as patients move between one hospital and another their microorganisms move together with them. This is particularly important for neonatal patients in an interconnected area, where they are frequently transferred from one NICU to another to undergo specialized procedures [16, 37]. This approach also allows for the coordination of procedures with the aim of optimizing assistance to the newborns [37]. With these goals, several neonatal networks have emerged in the world for the surveillance of care-related infections. The presence of an established surveillance program and of a working group allows for the identification of epidemiological changes in colonization trends and facilitates the implementation of effective control measures.

## Conclusions

This study shows the impact of implementing a coordinated strategy of control measures on MDR-GNB carriage in NICU patients and reveals its efficacy in obtaining a long-lasting reduction in the prevalence of MDR-GNB in an endemic setting.

The active surveillance program in the NICUs of the Palermo metropolitan area was useful in discovering the high-prevalence circulation of ESBL-KP in one NICU and to evaluate the efficacy of adopted measures. The implementation of preventive interventions proved to be effective, thanks to a cooperative and participatory approach between different professional profiles, all facing the problem of circulation and spread of antibiotic-resistant bacteria.

Periodic meetings were essential for sharing surveillance data, for increasing the awareness and relevance of the problem and for discussing critical points and possible solutions. Sharing of protocols, information and experiences within the NICU network of a metropolitan area could be a additional and important area of improvement.

## Data Availability

The datasets used and analysed during the current study are available from the corresponding author on reasonable request.

## Abbreviations

ESBL-KP: extended-spectrum β-lactamases-producing *Klebsiella pneumoniae*
MDR-GNB: multi-drug resistant Gram-negative bacteria
MDRO: multi-drug resistant organism
NICU: neonatal intensive care unit
PGFE: pulsed-field gel electrophoresis
Special characters: β
